# On the Improvement of Medicine

**Published:** 1838-07

**Authors:** 


ART. V.—*On the Improvement of Medicine. The Oration delivered before the Medical Society of London, at their Sixty-fifth Anniversary, March* 8, 1838. By THEOPHILUS THOMPSON, M.D., Physician to the Northern Dispensary, &c. *Published at the Request of the Society.* —*London*, 1838. 8vo. pp. 23.

WHATEVER share the improvement of medicine may have in the honour of producing such a result, the fact that the average duration of human life, in England and Wales, has considerably increased during the last half century, is one of very great importance. But there is reason to believe that medical science has really contributed to this end; the cure of some diseases, and the prevention of others, having been effected by its gradual advancement. As a proof of the advance made in the art of curing diseases, Dr. Thompson refers to the records of the puerperal hospitals; in one of which, in London, fifty years since, the average number of deaths was more than one in sixty; whereas, during the last fifty years, except during the prevalence of malignant fever, the mortality in such institutions has not been much more than one in three hundred. The effects of the prevention of disease are, of course, strongly illustrated by the great diminution of deaths from small-pox: the deaths now arising from that disease in London being about three hundred in a year, instead of five thousand. The banishment of the sea-scurvy is another of the legitimate triumphs of medicine. Dr. Thompson mentions, also, the improved treatment of the insane, and the greater number of recoveries from mental disorders; fewer of which are consequently fatal. The employment of the stethoscope; the diminution of surgical operations; and the greater attention paid to physical education, in consequence of the excellent works addressed to the public by physicians, are enumerated among the causes of the improved public health and more prolonged life. Dr. Thompson alludes to the investigations of living physiologists, as pointing to further useful results; especially to those of Dr. Todd, noticed in our Seventh number; and he makes an eloquent apology for homœopathic practice, without naming it, by observing (and we think justly,) that, "at the limits of visible anatomy, there lies another anatomy, whose phenomena are unrevealed;" and that "beneath the surface of our present physiology lie hid processes and laws, full of interest and wonders;" reasons, he thinks, for not turning contemptuously away from the asserted effects of minute agencies. The numerical method of Louis; the effects of the medical arrangements under the new Poor-Law Act; and the subject of medical education, are judiciously noticed in this oration; the language of which is throughout correct, spirited, and even elegant.

